# Psychometric properties of the forensic inpatient quality of life questionnaire: quality of life assessment for long-term forensic psychiatric care

**DOI:** 10.1080/21642850.2014.894890

**Published:** 2014-03-19

**Authors:** Ellen C.W. Vorstenbosch, Yvonne H.A. Bouman, Peter C. Braun, Erik B.H. Bulten

**Affiliations:** ^a^ Forensic Psychiatric Hospital Pompe Foundation, Nijmegen, The Netherlands; ^b^ Academic Centre for Social Sciences (ACSW), Radboud University, Nijmegen, The Netherlands

**Keywords:** quality of life, forensic psychiatry, assessment, psychometrics, questionnaire

## Abstract

A substantial group of forensic psychiatric patients require (life)long forensic psychiatric care. Instead of aiming at re-entry into society, treatment in long-term forensic psychiatric care (LFPC) is principally aimed at medical and psychiatric care and optimising quality of life (QoL). To assess QoL in LFPC, the influence of both the mental disorder and the restrictive context should be considered. Therefore, a new instrument was developed: the Forensic inpatient QoL questionnaire (FQL). The FQL is based on the results of concept-mapping with patients and staff within LFPC. The main purpose of this study is to evaluate the psychometric properties of the FQL. One hundred and sixty-three FQLs, filled out by 98 male long-term forensic psychiatric patients, were included for testing reliability and content validity. For testing construct validity, 53 patients additionally completed the World Health Organisation Quality of Life-Brief version and 50 of them the Affect Balance Scale. Outcomes indicate that the FQL has good psychometric properties. Fifteen of the 16 FQL domains showed adequate to good reliability (Cronbach's *α* range .69–.91) and 9 domains met the criteria for homogeneity. Content validity was demonstrated by exploratory factor analysis, which revealed a three-factor structure: social well-being, physical well-being and leave. Construct validity was supported by 59% correctly hypothesised inter- and intrascale Pearson's correlation coefficients. Good psychometric properties and its clinical-based development make the FQL a valid and useful instrument for QoL assessment in LFPC. The FQL could therefore contribute to evidence-based and more advanced treatment programmes in LFPC.

## 1. Introduction

Forensic psychiatric care is aimed at improving the mental health and reducing the risk of recidivism by mentally disordered offenders. In the Netherlands, treatment in a forensic psychiatric hospital can be imposed by a judge in case of diminished responsibility due to a mental disorder; if the perpetrator committed an offence that warrants a prison sentence of at least four years; and if a significant risk of serious re-offending is present (“terbeschikkingstelling”, tbs; Dutch Penal Code, art. 37a). Over the last decade, the duration of treatment in Dutch clinical forensic psychiatric facilities has increased considerably, leading to an average duration of over nine years.

Furthermore, there is a growing group of forensic psychiatric patients who have insufficiently benefited from the offered treatment methods and who are still deemed to pose a high risk for society (De Kogel, Verwers, & den Hartogh, 2005; Dienst Justitiële Inrichting, 2009; Expertisecentrum Forensische Psychiatrie, 2009; see also Salize & Dressing, 2007). A significant proportion of these patients may require long-term, potentially lifelong, forensic psychiatric care (Harty et al., 2004; Reed, 1997). In the Netherlands, a patient's main therapist can advise upon placement in a long-term forensic psychiatric facility if the risk of recidivism did not diminish sufficiently after two serious treatment attempts in two different forensic psychiatric hospitals. Subsequently, an independent committee of multi-disciplinary experts decides if placement in a long-term forensic psychiatric facility is considered adequate. Of the total Dutch forensic psychiatric population, about 10% currently resides in a specialised long-term forensic psychiatric care (LFPC) ward.

Typical for long-term forensic psychiatric patients is a complex psychopathology, non-compliance in therapy and/or poor learning abilities. Instead of treatment aimed towards re-entry into society, the main goal of long-term forensic psychiatry is to offer care in accordance with the rehabilitation principles. In LFPC, these principles entail psychiatric and medical care, acceptance of stay and optimising quality of life (QoL) within acceptable boundaries for society. The emphasis no longer lays on risk reduction and therefore on treatment of dynamic risk factors, but on QoL enhancement. In everyday clinical care, there is a need for practical guidance to achieve this.

Although there is a lack of consensus among researchers regarding the definition of QoL, on the whole it refers to an overall “sense of well-being and satisfaction experienced by people under their current life conditions” (Lehman, 1983). Some definitions focus on the subjective appraisal of life and its aspects (Bradburn, 1969; The World Health Organization Quality of Life [WHOQOL] Assessment, 1995), whereas others stress the assessment of objective indicators (such as health, living circumstances and income) to capture the construct of QoL (i.e. social indicators). However, in general QoL is considered as a multi-dimensional concept which encompasses both objective and subjective indicators (Bouman & Bulten, 2009; Fakhoury & Priebe, 2002; Katsching, 2006).

QoL assessment is increasingly used as a measure of treatment outcome and care evaluation in general psychiatry (Barry & Zissi, 1997; Lasalvia & Ruggeri, 2006). In clinical forensic psychiatry, however, QoL assessment is still uncommon (Bouman & Bulten, 2009; Rice & Harris, 1997) and few QoL studies have been conducted (van Nieuwenhuizen & Nijman, 2009; van Nieuwenhuizen, Schene, & Koeter, 2002; Saloppé & Pham, 2006a). The QoL instruments used in these studies are either generic or disease-specific. Although generic QoL instruments enable the comparison of outcomes with other populations, in LFPC generic instruments would assess irrelevant domains like access to public transport, while other more relevant domains such as contact with staff and other residents are absent (van Nieuwenhuizen, Schene, Boevink, & Wolf, 1997). Meanwhile, having a mental disorder influences many aspects of daily life in a more or less severe way (Diagnostic and Statistical Manual of Mental Disorders, version IV, DSM-IV-TR; American Psychiatric Association [APA], 2000), with a possible direct effect on QoL. Disease-specific instruments such as the QoL Interview (Lehman, 1988) and the Lancashire Quality of Life Profile (LQoLP; Oliver, Huxley, Priebe, & Kaiser, 1997) might therefore give a more accurate picture of the QoL of long-term forensic psychiatric patients. However, previous research in patients in long-term general psychiatry revealed that domains such as autonomy, self-esteem and coping abilities needed to be added to the LQoLP in order to assess QoL adequately (van Nieuwenhuizen, Schene, Koeter, & Huxley, 2001).

Nevertheless, in a forensic context, the suggested adaptations of the disease-specific LQoLP deficiently captured the total QoL concept (van Nieuwenhuizen, Schene, & Koeter, 2002; Swinton, Carlisle, & Oliver, 2001). The applicability of existing QoL instruments in a clinical forensic context has been questioned in several studies (van Nieuwenhuizen & Nijman, 2009; Swinton, Oliver, & Carlisle, 1999; Walker & Gudjonsson, 2000). Nonetheless, a QoL instrument that takes both the psychiatric and forensic implications into account had not been developed.

In order to employ the concept of QoL in LFPC, an integral model is needed that relates three components: (1) relevant objective indicators, (2) subjective evaluation and (3) directives to intervene. The first component, the objective indicators, should be assessed within the restricted setting with its corresponding deviant characteristics. In LFPC, a context where patients are forced to stay, aspects such as autonomy, lack of freedom, sense of control, restriction of movement and constraint of sexual relations, will get another connotation than in other, less restricted, settings (Coid, 1993; Mason, 1999; Mercier & King, 1994). The second component follows directly from the first, as it aims at capturing the subjective evaluation of the objective circumstances. The determinants of QoL should therefore be based on the values, conceptions, preferences and expectations of the patients (Mercier, 1994). Using determinants that are of importance for patients' perception of their QoL can offer directives to intervene and subsequently optimise patients QoL. A theoretical model that departs from the possibility to intervene is the Social Production Function (SPF) theory (van Bruggen, 2001; Lindenberg, 1996; Ormel, Lindenberg, Steverink, & Vonkorf, 1997). The SPF arose from the idea that individuals perform an active role in the enhancement of their own personal circumstances: towards optimal psychological well-being or an optimal QoL. To accomplish this, the individual should strive for two universal goals: *social and physical well-being*. Subsequently, these universal goals can be optimised by realisation of six instrumental goals. Where realisation of *status*, *behavioural conformation* and/or *affection* contributes to higher levels of social well-being, *internal comfort, external comfort and/or stimulation* contribute to higher levels of physical well-being. In order to enhance the level of well-being, different resources can be used. This implicates that individuals can substitute between resources when some resources are (temporarily) not accessible. Meanwhile, they are able to set up buffers for the negative effect of a sudden loss of resources (Nieboer & Lindenberg, 2002). Interventions can be tailored according to the SPF theory principles of substitution and buffering, and be adjusted if the realisation of certain goals is restricted by the individual's subjective and objective living conditions such as restriction of freedom or having a mental disorder.

The integrated model consisting of objective indicators, subjective evaluation and directives to intervene forms the bases of a newly developed instrument, the Forensic inpatient QoL questionnaire (FQL). However, before employing the FQL in care and translating the results into interventions which aim at the improvement of the circumstances of patients in LFPC, the psychometric properties of the FQL need to be established, specifically reliability and content and construct validity. The latter was done by exploring its relation with a generic QoL instrument (the World Health Organisation Quality of Life-Brief version, WHOQOL-Bref; WHOQOL Assessment, 1995; WHOQOL Group, 1998) and subjective psychological well-being (Affect Balance Scale, ABS; Bradburn, 1969).

## 2. Methods

### 2.1. Sample and procedures

The study was conducted at the long-term forensic care wards of the Pompe Foundation (the Netherlands). The long-term facility is situated on two separate locations: one on the venue of a prison where 48 long-term forensic psychiatric patients reside with a more high-risk profile. They are divided over six wards with 6 or 12 patients per ward. The majority of the patients (88) reside in a separate hospital. This last facility consists of 8 wards with 11 patients per ward. All patients are males with complex psychopathology. Of the 142 patients who stayed at the hospital during one time of the study, the vast majority was diagnosed with diagnoses on both Axis I and Axis II defined according to the DSM-IV-TR (APA, 2000). Only 6 patients (4.2%) did not have an Axis I diagnosis, and 15 patients (10.6%) did not have an Axis II diagnosis. Almost 60% of the population had a primary diagnosis on Axis II, mainly Personality Disorder Not Otherwise Specified (40 patients, 28%); and the most common diagnosis on Axis I was schizophrenia (39 patients, 28%).

In this study, the majority of patients suffered primarily from an Axis II disorder (*n* = 59, 60%) compared with those with a main diagnosis on Axis I (*n* = 39, 40%). Fifty-two participating patients (53%) were diagnosed with comorbid substance misuse. Subjects were convicted for the following offences: homicide (*n* = 28, 29%), violence (*n* = 25, 26%), sexual offence against adult (*n* = 16, 16%), sexual offence against minor (*n* = 22, 22%) or other offences such as arson (*n* = 7, 7%). Participants were between 31 and 71 years old, with a mean age of 47 years (SD = 9.7). On average, they stayed in the forensic psychiatric system for 163 (SD = 71) months, with an average stay in the long-term facility of 34 (SD = 23) months. Because patients are forced to stay in the LFPC, we asked them to grade their acceptance of stay on a 0–100 scale, 100 representing total acceptance. The average score on acceptance of stay was 38 (SD = 38).

Patients were contacted to participate by the day-to-day care members. The exclusion criteria were inability to complete the questionnaire due to psychotic episode and/or major chance of decompensation as judged by staff; insufficient mastery of the Dutch language or seclusion. In case patients suffered dyslexia, illiteracy or poor concentration, completion of the questionnaires was supported by a researcher, otherwise patients were asked to complete the questionnaires by themselves. The study was approved by the Ethics Committee of the hospital. Privacy of the patients was assured in accordance with the policy of the institution and analyses were conducted on anonymised data. After explanation of the purposes of the study, all participants gave informed consent.

This study is part of a continuing QoL project, different (amounts of) patients therefore participated in different stages of the study. Over a three-year period, 98 male long-term forensic psychiatric patients participated at a certain moment in the study (69%). During the first year, 53 patients completed the FQL and additionally the WHOQOL-Bref (WHOQOL Assessment, 1995; WHOQOL Group, 1998); one year later, 50 patients completed the FQL and the ABS (Bradburn, 1969); and in the last year, 60 patients completed the FQL. In total, 163 FQL questionnaires were completed (51%), 138 times patients refused to participate (43%) and 21 patients did not participate due to the exclusion criteria (6%).

### 2.2. Instruments

#### 2.2.1. Forensic inpatient quality of life questionnaire

The FQL is based on an integrated model consisting of three components: (1) objective indicators, (2) subjective evaluation and (3) directives to intervene. In order to assess the objective and subjective indicators in LFPC, a concept-mapping study was conducted among 40 LFPC patients and 30 staff members (Vorstenbosch, Bouman, Braun, & Bulten, 2010). Patients and staff's experiences and their perceptions on these experiences, which they considered important with regard to daily life in LFPC, were gathered using interviews. This resulted eventually in, respectively, 98 and 97 statements regarding QoL in LFPC. The statements were processed using a concept-mapping programme based on cluster analysis. The cluster analysis for patients and staff resulted in, respectively, 10 and 8 domains.

The items and domains resulting from the concept map formed the basis for the new setting- and disease-specific questionnaire: the FQL (Vorstenbosch, Bulten, Bouman, & Braun, 2007). Some domains, such as social relationships and affection, consisted mainly of objective indicators (e.g. having a girlfriend) which provided few means to intervene. These domains were therefore supplemented with adapted subjective items of the LQoLP (van Nieuwenhuizen, Schene, & Koeter, 1998) and items based on the SPF theory (Nieboer, Lindenberg, Boomsma, & van Bruggen, 2005).

The FQL contains 114 subjective and 17 objective items. Subjective items are assessed on a 100 mm Visual Analogue Scales (VASs), on which patients indicated their level of agreement with the specific item. This way scores ranged from 0 to 100, where 0 indicates no agreement at all and 100 complete agreement. Except the item “acceptance of stay”, the subjective items are clustered in 15 domains and 1 overall QoL measure consisting of 3 items. Table 1 summarises the characteristics of the FQL. Negatively formulated items were recoded in order to let high scores indicate a high QoL. Per domain, mean scores were calculated on a 0–100 scale.

**Table 1.** Descriptives and reliability per FQL domain.

*N *= 163	No. of VAS items	*n*	Mean (SD)	Cronbach's alpha	Mean inter-item correlation
(1) Activities	15	147	54 (20)	.89	.36
(2) Leave	2	112	34 (36)	.87	.77
(3) Residence	18	157	58 (22)	.91	.36
(4) Nutrition	3	160	47 (25)	.82	.61
(5) Hygiene	2	162	81 (23)	.72	.58
(6) Health	11	150	58 (19)	.77	.23
(7) Sexuality	2	146	32 (37)	.88	.79
(8) Social relations	8	156	64 (20)	.74	.27
(9) Other residents	6	156	54 (18)	.69	.28
(10) Daily staff	16	148	57 (19)	.89	.34
(11) Affection	14	156	58 (22)	.91	.41
(12) Self-actualisation	7	157	62 (17)	.70	.25
(13) Autonomy	4	159	63 (24)	.77	.45
*(14) Finances*	*2*	*161*	*64 (22)*	*.06*	*.03*
(15) Religion	2	150	63 (32)	.73	.58
(16) Overall QoL	3	159	44 (27)	.86	.67

Note: Domains with poor reliability (Cronbach's alpha <.70 and MIIC <.15 or >.50) are shown in italic typeface.

#### 2.2.2. WHOQOL-Bref

The construct validity of the FQL was assessed by comparing the FQL with the Dutch translation of the WHOQOL-Bref (Dutch translation by de Vries & van Heck, 1996). The WHOQOL-Bref is a self-report generic QoL questionnaire, consisting of 26 items each with a five-point Likert scale. The WHOQOL-Bref is considered reliable and valid among several psychiatric populations (Skevington, Sartorius, Amir, & The WHOQOL-Group, 2004; Trompenaars, Masthoff, van Heck, Hodiamont, & de Vries, 2005), including male adults in a forensic hospital (Saloppé & Pham, 2006b). Saloppé and Pham reported a satisfactory convergent and divergent validity and Cronbach's alphas between .77 and .79 for the subscales and .80 for the total WHOQOL-Bref. Following the criteria of the World Health Organisation, four domains, namely Physical health, Psychological health, Social relations and Environment, were calculated and transformed to a 0–100 scale (WHOQOL Group, 2000). Item 24 was excluded from the analyses since the participating patients are not in the position to make use of public transport.

#### 2.2.3. Affect Balance Scale

A Dutch translation of the ABS (Bradburn, 1969; Dutch translation as used in the Dutch LQoLP of van Nieuwenhuizen et al., 1998) was selected because of its association with QoL in previous studies (Baker & Intagliata, 1982; Oliver et al., 1996; Ryff, 1989). The ABS is a self-report questionnaire measuring overall psychological well-being. The ABS consists of 10 items, with 5 items measuring positive affect and 5 items measuring negative affect. Each item is scored on a seven-point Likert scale. Bradburn (1969) reported test–retest reliabilities for positive and negative affect of, respectively, .83 and .81. Regarding validity, small correlations between positive and negative affect were found and factor analysis underscored the structure of two dimensions on the ABS. Total scores were computed separately for the positive affect and negative affect. Scores on the negative items were reversed, so that higher negative scores indicate more negative affect.

### 2.3. Statistical analyses

Data from three-assessment moments were used to evaluate internal consistency and content and construct validity.

Missing data were detected by conducting missing value analysis. Data were not missing structurally, but some patients interpreted a few items as being not applicable to them. In case a subscale was missing more than one item, no mean score was calculated for the particular subscale.

#### 2.3.1. Reliability

Reliability was examined by calculating internal consistency and mean inter-item correlation (MIIC). Cronbach's *α* was calculated as a measure of internal consistency. An alpha coefficient of ≥0.70 was considered acceptable (Nunnaly, 1978). In addition, MIICs were calculated in order to assess homogeneity of the FQL and its domains (Clark & Watson, 1995). An MIIC between .15 and .50 is desirable (Briggs & Cheek, 1986). Subscales with poor internal consistency and homogeneity were not entered in the validity study.

#### 2.3.2. Validity

Two types of validity were tested. Content validity was examined by performing an exploratory factor analysis in which all FQL domains were entered except the measure for overall QoL. The factor analysis was performed using a principal component analysis with varimax rotation and eigenvalues exceeding 1. Construct validity was examined by calculating the interscale correlations of the FQL with the WHOQOL-Bref and the ABS and by examining the strength of the correlations within the FQL domains. A priori hypotheses about the strength and direction were formulated for both intrascale and interscale correlations. A high percentage of correct predictions between questionnaires indicates a stronger support for construct validity. Pearson's correlations of .10–.30 were considered weak, .30–.50 moderate and  >.50 were defined as strong (Cohen, 1988).

Regarding the intrascale correlations, we expected all FQL domains to correlate moderately to strongly with overall QoL. Furthermore, we expected moderate to strong correlations between domains with a social component such as social relations, affection and other residents. Likewise, we expected domains with an environmental component to correlate moderately to strongly, such as residence, activities, nutrition, hygiene and autonomy. Because daily staff intervenes in almost all daily aspects, we expected daily staff to show moderate to strong correlations with the majority of the FQL domains.

The interscale correlations were expected to be moderate to strong between the WHOQOL-Bref subscales and overall QoL of the FQL. Between the subscales of the WHOQOL-Bref and its FQL counterparts, we expected moderate to strong correlations. That is, physical health with health, psychological health with health and self-actualisation, social relations with social relations, sexuality and affection, and environment with residence. Because leave, other residents, daily staff and autonomy are not assessed by the WHOQOL-Bref, we expected weak correlations between these domains of the FQL and the WHOQOL-Bref domains. With regard to the ABS and overall QoL, we expected moderate to strong positive correlations for positive affect, and moderate to strong negative correlations for negative affect (Bradburn, 1969; Oliver, Huxley, Bridges, & Mohamad, 1996; Skevington & Wright, 2001). Based on Bradburn's findings, we expected positive affect to correlate moderately to strongly with activities, social relations, other residents and self-actualisation, and negative affect to correlate moderately to strongly with residence and autonomy. With daily staff intervening in almost all daily aspects, a negative as well as a positive affect may be provoked. We expected therefore daily staff to show moderate to strong correlations with both dimensions of the ABS.

## 3. Results

### 3.1. FQL domain descriptive statistics

Patients in LFPC were most satisfied with their hygiene (*m* = 81) and least happy on the domain sexuality (*m* = 32). Their mean overall QoL was just below the possible midpoint (*m* = 44; SD = 27; Table 1).

Assessment of psychometric properties is given as follows.

#### 3.1.1. Reliability

The internal consistency and the homogeneity of the FQL can be considered adequate, with Cronbach's *α* coefficient of .88 and an MIIC of .37. The domain finances showed Cronbach's *α* of .06 and an MIIC of .03, and are therefore considered unreliable and excluded for validity analysis. The domain other residents gave Cronbach's *α* of .69, but showed an acceptable MIIC of .28 and remained included. The remaining FQL domains showed acceptable to high internal consistencies with Cronbach's *α* coefficients ranging between .70 and .91 (Table 1). The MIIC values of these domains ranged from .23 to .79. Six domains did not meet the criteria for homogeneity: leave, nutrition, hygiene, sexuality, religion and overall QoL.

#### 3.1.2. Exploratory factor analysis

**Figure 1.**
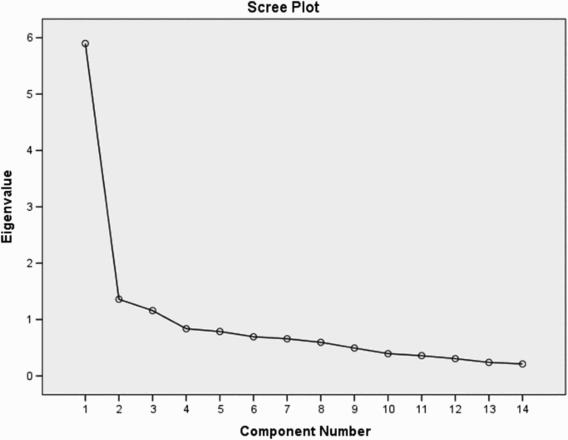
Screeplot of the eigenvalues for factor analysis of the FQL.

The factor analysis showed that a three-component solution would provide the best groupings of domains with 60% of the variance explained, with Factor 1 contributing 30%, Factor 2 21% and Factor 3 9% of the variance, respectively (Figure 1).

The first factor contained health, residence, activities, hygiene, religion, nutrition, daily staff and self-actualisation. The second factor comprised sexuality, social relations, affection, other residents and autonomy, and the third factor leave (Table 2).

**Table 2.** Exploratory factor analysis: varimax rotation of three-factor solution for FQL domains (*N *= 163).

	Factor 1	Factor 2	Factor 3
Health	**.75**	.34	.11
Residence	**.72**	.31	.14
Activities	**.71**	.42	.23
Hygiene	**.69**	.04	−.36
Religion	**.68**	.13	−.11
Nutrition	**.68**	.04	.26
Daily staff	**.62**	.44	.19
Self-actualisation	**.52**	.31	.13
Sexuality	−.00	**.70**	.26
Social relations	.17	**.70**	−.33
Affection	.35	**.68**	.07
Other residents	.32	**.66**	−.22
Autonomy	.51	**.57**	.29
Leave	.19	.01	**.85**
% of variance explained	29.8%	20.6%	9.8%

Note: Domains are in boldface to indicate on which factor they had the highest loading.

For the three obtained factors, the mean scores were the following: Factor 1, *M* = 60 (SD = 16); Factor 2, *M* = 54 (SD = 18); and Factor 3 m *M* = 34 (SD = 36).

#### 3.1.3. Construct validity

Overall QoL correlated significantly (*p* < .01) with most domains (Table 3), on a range from .23 to .62. Strong correlations were found with activities, residence, health, daily staff and autonomy; whereas weak correlations were found with sexuality, other residents and religion. No significant correlations were found with leave, hygiene and social relations. Other domains correlated moderately with overall QoL. In accordance with our expectations, FQL domains with a social component, that is social relations, other residents and affection, showed moderate to strong intrascale correlations (ranging from .41 to .53, *p* < .001). Residence showed moderate to strong relations with the environmental components activities (*r* = .64, *p* < .001), nutrition (*r* = .47, *p* < .001), hygiene (*r* = .49, *p* < .001) and autonomy (*r* = .57, *p* < .001). This was in line with our expectations. Low, and in cases non-significant, correlations (ranging from .01 to .28) were found between leave and the other FQL domains. Except from sexuality and leave, daily staff correlated moderately to strongly at a *p* < .001 level with all FQL domains. Seventy-eight per cent of the Pearson's correlation coefficients were appropriately a priori hypothesised.

**Table 3.** Intrascale Pearson's correlations of the FQL (*N *= 163).

	1	2	3	4	5	6	7	8	9	10	11	12	13	14
(1) Activities	–													
(2) Leave	.29^b^	–												
(3) Residence	**.64**^b^	.25^a^	–											
(4) Nutrition	**.55**^b^	.28	.47^b^	–										
(5) Hygiene	.35^b^	−.05	.49^b^	.30^b^	–									
(6) Health	**.70**^b^	.22	**.58**^b^	**.50**^b^	.41^b^	–								
(7) Sexuality	.35^b^	.14	.30^b^	.16	.09	.24^a^	–							
(8) Social relations	.36^b^	−.14	.27^b^	.12	.20	.39^b^	.28^b^	–						
(9) Other residents	.45^b^	−.02	.46^b^	.19	.31^b^	.36^b^	.31^b^	.41^b^	–					
(10) Daily staff	**.60**^b^	.26^a^	**.54**^b^	.35^b^	.30^b^	**.68**^b^	.22	.36^b^	.41^b^	–				
(11) Affection	.46^b^	.15	.37^b^	.32^b^	.25^a^	.48^b^	.36^b^	.41^b^	**.53**^b^	**.51**^b^	–			
(12) Self-actualisation	.45^b^	.07	.44^b^	.34^b^	.26^a^	.44^b^	.19	.16	.24^a^	.47^b^	.49^b^	–		
(13) Autonomy	**.64**^b^	.28^a^	**.57**^b^	.41^b^	.33^b^	**.53**^b^	.41^b^	.39^b^	.42^b^	**.61**^b^	.48^b^	**.52**^b^	–	
(14) Religion	**.52**^b^	.10	.39^b^	.36^b^	.37^b^	**.51**^b^	.16	.24^a^	.32^b^	.45^b^	.33^b^	.29^b^	.29^b^	–
(15) Overall QoL	**.61**^b^	.22	**.54**^b^	.43^b^	.15	**.55**^b^	.26^a^	.12	.23^a^	**.53**^b^	.37^b^	.45^b^	**.55**^b^	.27^a^

Note: Strong correlations (*r *≥ .5) are shown in bold typeface.

^a^Correlations are significant at the .01 level (two-tailed).

^b^Correlations are significant at the .001 level (two-tailed).

To further assess construct validity, the correlations between the FQL and the WHOQOL-Bref, and the ABS were calculated (Table 4). These outcomes showed that 59% (22) of the hypothesised correlations were correct. Overall QoL showed strong correlations with only two of the WHOQOL-Bref domains, psychological health (*r* = .46, *p* < .01) and environment (*r* = .59, *p* < .001). Moderate to strong correlations were found between domains of the FQL and its WHOQOL-Bref equivalence: health with physical health (*r* = .52, *p* < .001), self-actualisation with psychological health (*r* = .44, *p* < .01), sexuality and affection with social relations (*r* = .49, *p* < .01; *r* = .64, *p* < .001) and residence with environment (*r* = .53, *p* < .001). Low non-significant correlations were found with leave (*r* = .00 to .13) and other residents (*r* = .17 to .28). False predictions were made with regard to health with psychological health and social relations with social relations, both showed non-significant correlations. Daily staff and autonomy showed moderate to strong correlations with almost all WHOQOL-Bref domains, respectively, ranging from .38 to .50 and .38 to .56.

**Table 4.** Construct validity as measured by Pearson's correlation between FQL domains and the WHOQOL-Bref (*n *= 48), and the ABS (*n* = 43).

	WHOQOL-Bref				ABS	
	Physical health	Psychological health	Social relations	Environment	Positive affect	Negative affect
(s1) Activities	.42^a^	.42^a^	.56^b^	.73^b^	**.62**^b^	**−**.29
(2) Leave	**.00**	**−.04**	**.06**	**.13**	.10	**−**.55
(3) Residence	.31	.37^a^	.17	**.53**^b^	.53^b^	**−**.37
(4) Nutrition	.06	.30	.22	.31	.44^a^	**−**.39^a^
(5) Hygiene	.28	.25	.16	.22	.42^a^	**−**.33
(6) Health	**.52**^b^	.34	.46^a^	.44^a^	.62^b^	**−**.40
(7) Sexuality	**−**.02	.32	**.49**^b^	.35	.34	**−**.06
(8) Social Relations	.37	.33	.36	.29	.29	**−**.26
(9) Other residents	**.28**	**.17**	**.26**	**.21**	**.42**^a^	**−**.03
(10) Daily staff	.38^a^	**.36**	.42^a^	.50^b^	**.65**^b^	**−**.33
(11) Affection	.41^a^	.42^a^	**.64**^b^	.45^a^	.28	**−**.05
(12) Self-actualisation	.39^a^	**.44**^a^	.28	.25	**.45**^a^	**−**.06
(13) Autonomy	.40^a^	.38^a^	.43^a^	.56^b^	.58^b^	**−**.20
(14) Religion	.31	.23	.18	.27	.36	**−**.17
(15) Overall QoL	.35	**.46**^b^	.34	**.59**^b^	**.66**^b^	**−.43**^a^

Note: Appropriately a priori predicted correlations are shown in bold typeface.

^a^Correlations are significant at the .01 level (two-tailed).

^b^Correlations are significant at the .001 level (two-tailed).

In line with our expectations, overall QoL showed moderate to strong correlations with both dimensions of the ABS (*r* = .66, *p* < .001 with positive affect and *r* = −.43, *p* < .01 with negative affect). Activities (*r* = .62, *p* < .001), residence (*r* = .53, *p* < .001), nutrition (*r* = .44, *p* < .01), health (*r* = .62, *p* < .001), other residents (*r* = .42, *p* < .01), daily staff (*r* = .65, *p* < .001), self-actualisation (*r* = .45, *p* < .01) and autonomy (*r* = .58, *p* < .001) were moderately to strongly related with positive affect. Nutrition (*r* = −.39, *p* < .01) showed a moderate correlation with negative affect.

Although not predicted, strong relations were found between activities and the WHOQOL-Bref domains social relations (*r* = .56, *p* < .001) and environment (*r* = .73, *p* < .001), and positive affect of the ABS (*r* = .62, *p* < .001). Autonomy correlated strongly with the WHOQOL-Bref domain environment (*r* = .56, *p* < .001).

## 4. Discussion

In this study, the reliability and validity of a newly developed instrument for QoL assessment in LFPC were examined. The results showed that the disease- and setting-specific FQL has acceptable to good psychometric qualities. Good internal consistency and homogeneity were found for the total FQL and for 9 of the 15 domains. One domain, other residents, showed a moderate Cronbach's *α* and needs further investigation. The domain, finances, lacked both internal consistency and homogeneity and should be revised. Five other domains, which contain only two or three items, showed poor homogeneity. This means that the items in these domains are either highly redundant or do not show enough differentiation (Clark & Watson, 1995).

Conceptually, content validity was guaranteed by the instrument's construction process. The content of the FQL was derived from the results of a concept-mapping within LFPC (Vorstenbosch et al., 2010). Furthermore, exploratory factor analysis revealed a three-factor structure, which explained 60% of the variance in overall QoL. The first factor shows similarities with the SPF goal of physical well-being. The second factor has resemblances with the social well-being component of the SPF. The third factor was centred around the FQL domain leave.

Construct validity of the FQL was demonstrated by correctly predicted a priori hypotheses on intra- and interscale levels. Overall QoL related to all FQL domains except leave, hygiene and social relations. Besides, no high interscale correlations (Cohen, 1988) for the FQL and, respectively, the WHOQOL-Bref and the ABS were found, which would have implied that the used instruments or tested domains are interchangeable. Two WHOQOL-Bref domains were related to overall QoL: psychological health and environment. In line with our hypothesis, both positive and negative affect were related to overall QoL. Furthermore, strong relations were found between several FQL domains and positive affect, whereas only nutrition was related to negative affect.

Given that leave is not an issue in everyday life of other populations, this domain has never been included in QoL studies to our knowledge (Mason, 1999). The absence of a relation between overall QoL and leave is remarkable, since the results revealed this domain as a substantially contributing aspect to QoL. Further research is needed to clarify if leave is an aspect of QoL in LFPC or the domain's content needs reconsideration, the more since its MIIC was high.

Consistent with a study by Bouman, de Ruiter, and Schene (2008), no relation was found between social relations and overall QoL. This might suggest that forensic patients differ from other populations regarding this aspect. Both severe psychopathology and many years in forensic services complicate relating to others and may often result in losing contact with family and/or friends. Revision of the items, adding a plain explanation for patients who do not have contact with family and/or friends, might give a more accurate representation of the patients' perception on their social relations.

A strong positive relation was found for daily staff with overall QoL, affection and positive affect. This underlines the assumption that staff play an important role in everyday life of long-term forensic psychiatric patients. Although this has been concluded by several authors (Boevink, Wolf, van Nieuwenhuizen, & Schene, 1995; Coid, 1993; Mason, 1999; Oliver et al., 1996), it has not been included in QoL assessment in general psychiatry (van Nieuwenhuizen et al., 2001; Oliver et al., 1997). Because of the importance of this aspect, it should be explored in more detail in future research, also in general psychiatry.

The current study has a number of obvious limitations. First, the results of this study might be less generalisable to other forensic and/or psychiatric populations because the sample size was relatively small and patients from only a single long-term forensic psychiatric centre were involved, although the vast majority of Dutch LFPC patients reside in that facility. Since the start of the project, the FQL has been translated into German and English and has been used in German and English forensic psychiatric hospitals. Data gathered in those facilities, together with Dutch data, will in the future possibly lead to further refinements in the instrument. In addition, the participants formed a mixed group of patients with different levels of severe psychopathology and/or different diagnoses and subjects spend a high average of years in forensic psychiatric care. Second, the analyses rely exclusively on self-reports. The forced context in which the participants reside might cause frustration and resistance against the system, which might reflect in a general negativity that does not necessarily coincide with reality. Future research should include the development of a proxy version for staff and a checklist for more objective indicators. It will be important to analyse the relationship between the FQL of the patient, the FQL of the mentor and more objective indicators. Third, more validation work is needed to assess the FQL's responsiveness and sensitivity. Finally, the population of Dutch long-term forensic psychiatric patients, although growing, is limited and the amount of participating patients will therefore remain inevitably small. Cross-cultural adaptation and validation will therefore be necessary.

### 4.1. Conclusion

The FQL seems an appropriate QoL instrument for the population of long-term forensic psychiatric patients. Although further study is needed, the promising psychometric results and the content deriving from clinical practice make the FQL a valid and useful QoL instrument for assessment in this forensic psychiatric subpopulation. Both the major influence of having a mental disorder and the restrictions of the deviant context are taken into consideration, which distinguishes the FQL from other QoL instruments such as the WHOQOL-Bref. Along with the substitution and buffer principles of the SPF theory, the FQL is an appropriate and applicable instrument for everyday LFPC. For forensic patients who are incarcerated for a (life)long period, the FQL provides opportunities to enhance their QoL. Meanwhile, research into QoL will contribute to evidence-based practice and more advanced treatment programmes in LFPC.
